# The Relationship Between Refugee Health Status and Language, Literacy, and Time Spent in the United States

**DOI:** 10.3928/24748307-20201109-01

**Published:** 2020-12-11

**Authors:** Iris Feinberg, Mary Helen O'Connor, Ashli Owen-Smith, Michelle Mavreles Ogrodnick, Richard Rothenberg

## Abstract

**Background:**

There are 3 million refugees living in the United States today whose health and wellbeing may be diminished by not being able to understand and use health information. Little is known about these barriers to health in multiethnic refugee communities.

**Objective:**

This present study examined (1) the relationship between English proficiency, health literacy, length of time in the US, and health status; and (2) differences in poor health status caused by limited English proficiency and low health literacy individually and in combination to better understand which barriers might be addressed by improving refugee health.

**Methods:**

Refugees (*N* = 136) age 18 to 65 years were recruited using health clinics and refugee resettlement agencies. Survey questions included demographics, health status, health literacy, English language proficiency, social determinants of health, and barriers to getting health care. Interpreters were used as necessary. We used a cross-sectional study with purposeful sampling.

**Key Results:**

There is a high correlation (Pearson's *r* = 0.77) between health literacy and English proficiency; they were moderately correlated with health status (*r* = 0.40 and 0.37, respectively). Length of time in the US only modestly correlated with health status (*r* = 0.16). Health literacy and English proficiency taken individually were strong predictors of health status (health literacy odds ratio [OR] = 4.0; 95% confidence interval [1.6–9.9], English proficiency OR = 3.6, confidence interval [1.5–9.0]) but not significant. Their interaction, however, was significant and accounted for most of the effect (log odds for interaction = 1.67, OR = 5.1, *p* < .05).

**Conclusions:**

English proficiency and health literacy individually and in combination facilitate poor health and present health-related barriers for refugees. Length of time in the US for refugees may not correlate with health status despite studies that suggest a change in health over time for the larger immigrant population. **[*HLRP: Health Literacy Research and Practice*. 2020;4(4):e230–e236.]**

**Plain Language Summary:**

The combined effects of limited English proficiency and low health literacy can create significant barriers to good health outcomes in refugee populations. Length of time in the US for refugees may not correlate with health status despite studies that suggest a change in health over time for the larger immigrant population.

More than 71 million people worldwide have been forcibly displaced due to ethnic violence, natural disasters, failed governments, civil wars, and political oppression and are unable to live safely in their own countries (United Nations Human Rights Council [UNHRC], 2019). Most of these people are displaced within their own countries; others are migrants who leave their homes to live in another country for economic reasons. Close to 20 million others fear persecution or cannot seek protection from their own governments, so they seek asylum in other countries and enter with protections under international law and special refugee rights (UNHRC, 2019). More than 3 million refugees have been settled in the United States since 1980, and received resettlement resources that last up to 1 year and may include health insurance, living stipends, English language classes, and job training (National Immigrant Forum, 2019). Even with this assistance, the refugee community continues to struggle with high rates of mortality and morbidity, trauma-induced stress, poor access to basic health care, and poor health status.

Little is known about barriers relating to health care for refugees who live in multiethnic communities; most studies have examined single-ethnic community needs, perceptions of health providers, or comparisons of immigrant versus host populations (Drewniak et al., 2017; Fiddian-Qasmiyeh, 2016; Murphy et al., 2019). Clarkston, Georgia, is a multiethnic community (Bloemmart & Bacchus, 2013) where more than 17,000 refugees who speak 60 different languages have been settled since 2004. Health care is provided by two free clinics that are run by community volunteers, several sliding-scale clinics where fees are charged based on income level, a health center run by the county under the aegis of the statewide Department of Public Health, and a local hospital. Cultural and language discordance exacerbate the challenges that refugees face in accessing health care services, which are basic, fragmented, and duplicative with needs like vision, dentistry, and mental health services left unaddressed (DeKalb County, 2015; Refugee & Immigrant Health and Wellness Alliance, 2019). Need for care is complicated by social determinants such as language proficiency, literacy skills, income, transportation, the built environment (housing, sidewalks), and culture (Adler et al., 2016).

Limited English proficiency (LEP) is a social determinant that creates a barrier to health care (Coren et al., 2009; Gadon et al., 2007; Murphy et al., 2019); patients may not understand diagnosis and care plans, which results in delays in preventive services, missed office visits, unmanaged acute conditions, and exacerbation of chronic conditions (Coren et al., 2009; Jacobs et al., 2004; Morris et al., 2009). As language proficiency increases, so do most facets of social integration including feelings of agency, belonging, and independence (Court 2017). Language-concordant clinical staff and interpreters may ease burdens associated with LEP, but oral instructions and written materials are often delivered only in English, and are inaccessible to refugees with LEP (Diamond et al., 2019; Jacobs et al., 2004; US Department of Health and Human Services, Office of Disease Prevention and Health Promotion, 2020). The language of health care, whether written or oral, has its own formal, jargon-laden, and colloquial register, and although it can be difficult for anyone to understand and use health information, understanding the language of health care is particularly complex for people with language, literacy, or cultural barriers (Berkman et al., 2011; DeWalt et al., 2004; Peerson & Saunders, 2009).

There are 3 million refugees living in the US today whose health and wellbeing may be diminished by not being able to understand and use health information. Our first research question examines relationships between health status, English proficiency, health literacy, and length of time in the US controlling for age and gender. Our second research question examines differences in poor health status by LEP and low health literacy individually and in combination. The present study examined these barriers that may be affected through resettlement efforts and policy change (Capps et al., 2015; Jacobsen & Fratzke, 2016). Further, English proficiency and adequate health literacy are related to improved health outcomes in the general population (DeWalt et al., 2004); we posit that this is also true for refugees living in a multiethnic community.

## Methods

### Sample

Adult refugees who live in and around Clarkston, GA, were recruited using several health clinics and refugee re-settlement agencies; of the 150 people who were approached, 136 agreed to be in the study. All participants were born overseas and were age 18 years or older. The consent form was read to participants and interpreters were available to assist with informed consent and survey administration. Interpreters were required for 110 participants with the primary languages being Swahili, Arabic, and Burmese. One researcher was fluent in Swahili and French and one was fluent in Arabic so they assisted with consents and surveys in addition to student volunteers and clinic and agency volunteers and staff. The Georgia State University Institutional Review Board approved this study.

### Measures

The 58-question survey included questions about demographics, health, health literacy, English language proficiency, social determinants of health, and barriers to getting health care in the US. Demographic questions were age, gender, nativity, length of time in US, years of schooling, marital and family status, employment, and income and sources of income. Questions measuring English language proficiency were derived from the Programme for the International Assessment of Adult Competencies, an Organization for Economic Cooperation and Development (2016) household survey that measures literacy, numeracy, and digital problem solving. To measure health, we used questions derived from the Centers for Disease Control and Prevention (2017) Behavioral Risk Factor Surveillance System on health status, use of preventive measures, disease status, and use of health facilities. Health literacy was measured using the 3-questions Brief Health Literacy Screener (Chew et al., 2004). Questions used to measure social determinants were derived from the Health Leads (2016) Screening Toolkit that was developed using the Institute of Medicine, The National Association of Community Health Centers, and Centers for Medicare and Medicaid Services guidelines. The survey was delivered orally in English and interpreters were available to assist participants. Each survey was 1 hour, and each participant received $25. Interpreters were available either on site or on the telephone; interpreters were paid $25 for being part of the study except for those who were clinic or agency staff and could not accept remuneration for their assistance.

Specific to our study, a Health Literacy Scale was derived from the three health literacy questions: (1) Do you have someone to help you read hospital or clinic materials?; (2) Do you have problems learning about your medical condition because you have trouble understanding written information?; and (3) Do you have trouble filling out medical forms by yourself? These questions contained points from the Brief Health Literacy Screener (Chew et al., 2004), a general health literacy measure that is easy and quick to administer (1.5 minutes) and has been validated against other health literacy measures in various populations to detect inadequate or marginal health literacy (Chew et al., 2004; Chew et al., 2008; Wallace et al., 2007; Wallace et al., 2006). The resultant Health Literacy Scale variable is dichotomized to low health literacy (need help *all*, *most*, or *some of* the time) and adequate health literacy (*do not need help*). An English Proficiency Scale was derived from the English proficiency questions “How well would you say you read/write/ speak English?” These were dichotomized into *not at all/ not well* and *well/very well*. Length of time in the US was measured as less than or more than 2.5 years, which is our sample median; we chose the median because our responses had extreme outliers that, even when transformed was still significantly skewed, and because, within a few years of arrival, many refugees have improved English skills and are in the workforce (US Department of Health and Human Services, Office of Refugee Resettlement Fiscal, 2016).

### Statistical Methods

We used SAS 9.4 for data analysis. Descriptive statistics included means, standard deviations, frequencies, and chi-square calculations. Logistic modeling used bivariate dependent and independent variables to assess the individual and combined importance of English proficiency, health literacy, and length of time in the US with gender and age as covariates.

## Results

### Characteristics of the Study Population

Participants (*N* = 136) came from 22 different countries; 88% were from nine countries: (the Democratic Republic of Congo [*n* = 37], Burma [*n* = 27], Afghanistan [*n* = 10], India [*n* = 10], Iraq [*n* = 9], Ethiopia [*n* = 9], Tanzania [*n* = 7], Syria [*n* = 6], Burundi [*n* = 5]). Participants' average age was 39 years and one-third of the participants were male. Slightly more than one-half have been in the US for 2.5 years or less, and 40% were employed. Educational attainment was less than an average of 9 years. Most participants were below the poverty level, and less than 30% had health insurance. Approximately two-thirds had LEP and low health literacy (**Table 1**).

### Role of Health Literacy, English Proficiency, and Time in the US

There was a statistically significant, strong positive correlation between health literacy and English proficiency, *r*(134) = .77, *p* < .05, and a moderate positive correlation between health literacy and health status *r*(134) = .40, *p* < .05 and English proficiency and health status *r*(134) = .37, *p* < .05 (**Table 2**).

A logistic regression was performed to ascertain the effects of health literacy, English proficiency, length of time in the US, the interaction between health literacy and English proficiency, gender (female as referent), and age on health status. In individual regressions, both health literacy and English proficiency had substantial point estimates odds ration 4 (95% confidence interval [1.6-9.9]) and odds ratio 3.6 (95% confidence interval [1.5-9]) but neither was significant. A binomial logistic regression was performed to ascertain the effects of health literacy, English proficiency, the interaction of health literacy and English proficiency, length of time in the US, age, and gender on health status. The model was statistically significant X^2^ (6) = 37.238, *p* < .05. Of the six predictor variables that were included in the model, only two were significant: the interaction between health literacy and English proficiency and age (as shown in **Table 3**). Those with inadequate health literacy and LEP were 5.08 times more likely to have poor/fair health, and as age increases health status decreases by 4%.

## Discussion

This study has two key findings: (1) both English proficiency and health literacy are individual barriers to poor health but some combination of the two may be more indicative of health-related challenges for refugees; and (2) length of time in the US for refugees may not correlate with health status despite studies that suggest a change in health over time for the broader immigrant population.

English proficiency continues to be one of the most challenging aspects of resettlement for refugees. LEP is a widely documented barrier to health care and can have a compounding effect on other social determinants like income, employment, and transportation (Adler et al., 2016; Coren, et al., 2009; US Department of Health and Human Services, Office of Disease Prevention and Health Promotion, 2020). A gap exists in understanding the relationship between health status and English proficiency among the general population with LEP; the Spanish-speaking community has been the most frequently studied (Sentell & Braun, 2012). Refugees have generally not been represented in this work even though they have a high prevalence of LEP. One reason for this gap is that refugees come to the US speaking multiple languages and are not a homogenous group. Participants in our study came from a variety of countries (primarily the Democratic Republic of Congo, Burma, Afghanistan, India, Iraq, Ethiopia, Tanzania, Syria, and Burundi) and represent multiple ethnic groups with distinct languages and cultures. Collecting data across multiple languages and cultures is complex and time consuming, and there is no current survey that accommodates these differences.

We found that refugees who are more proficient in English also report stronger health literacy skills. Health literacy is a specialized form of literacy that includes reading, writing, speaking, and listening skills along with an ability to engage in dialogue with a health care provider (Feinberg et al., 2018). People with LEP are also likely to have low health literacy; there is a direct association between low health literacy and poor health status (Paasche-Orlow & Wolf, 2007). Adults with low health literacy are less likely to manage their health when sick, seek health information, and know about preventive behaviors (Feinberg et al., 2019). Health literacy is not an individual trait because it also depends on how health information is delivered. The language of health care uses medical jargon, a direct communication style, and is governed by regulatory requirements that increase the health literacy demands on patients (Cayton, 2006; Roter, 2011). Adults with low health literacy may also struggle due to lack of knowledge about how the body works, language skills, and intercultural communication issues (Baker, 2006; Feinberg et al., 2017).

LEP and low health literacy often co-occur but are less often studied together (Sentell & Braun, 2012). There are widespread data on Spanish-speaking immigrants, but none that study the relationship between language proficiency and health literacy for a diverse refugee population who are often lumped into the racial or ethnic category “other” if included at all in research (Floyd & Sakellariou, 2017). LEP and low health literacy combined decrease access to care, patient comprehension, and patient adherence (Wilson et al., 2005). Our study helps to fill this gap in refugee health research by considering the relationship between language skills, health literacy, and health status.

We know that adults with low health literacy may not understand how the body works or understand health terms; for refugees, the problem may be further compounded by discordant cultural beliefs—health and wellbeing viewed through refugee cultural beliefs may be dissimilar to those of Western medicine (Feinberg et al., 2017). The ability of health professionals to impart important biological, diagnostic, and care information can be complicated due to this confluence of patient LEP, low scientific knowledge, discordant beliefs with Western medicine, and low health literacy. In some cultures, there are no words for familiar Western medical terms (Refugee & Immigrant Health and Wellness Alliance, 2019) and traditional or alternative health practices may be common in refugee home countries or cultures. The biomedical model of disease etiology may be completely antithetical to a refugee's beliefs about health and wellness.

We found no significant relationship between length of time in the US and health. The “healthy immigrant effect” theory posits that the longer immigrants stay in the US, the worse their health becomes as they assimilate; health status equalizes between immigrant and nonimmigrant levels within 10 to 20 years (Markides & Rote, 2019). Most research on this phenomena center on broad immigration patterns that include those migrating to the US for employment with sought-after occupational skills and with a primary focus on the 19.7 million immigrants from Latin American who make up 44% of the total immigration population (Markides & Rote, 2019). Many refugees come to the US with experiences of psychological and physical stress, low levels of occupational skills and from distant countries with culturally and linguistically dissonant backgrounds; they also represent many diverse subpopulations rather than the more heterogeneous groups that come from Latin America (Markides & Rote, 2019). There are few individual-level data sets collected in diverse refugee communities, and no nationally coordinated data collection effort; both are needed to understand the needs, diversity, and approaches to refugee integration in the US.

Regardless of length of time in the US, refugees with both LEP and low health literacy are a particularly vulnerable group and have a high prevalence of poor health (Kandula et al., 2007; Murphy et al., 2019; Sentell & Braun, 2012). Our study shows that some combination of LEP and low health literacy combined may have the most deleterious effect on health status. These findings demonstrate the importance of unraveling the effects of LEP and low health literacy on health status, particularly for populations who are rarely studied but who report high levels of poor health. The ability to obtain, understand, and use health information is a key tenet of health literacy; for refugees, these skills may not be developed and may contribute to poor health. Thus, delivering understandable and useable health information must become the responsibility of those who provide that health information. Supported by a body of evidence substantiating its effectiveness, culturally and linguistically appropriate standards are health literate communication techniques that can improve refugee health by reaching patients where they are and providing information that is understandable and usable (US Department of Health and Human Services, Office of Minority Health, 2019). Analysis of language-concordant patient-provider communication may help reduce the impact of LEP and low health literacy, but these relationships are also complex and do not necessarily consider the complications of refugee resettlement and integration (Morris et al., 2009). Development of health education and promotion materials sensitive to refugee health, language, and health literacy needs could contribute to improved health outcomes for the 3 million refugees currently settled in the US. Consideration of language skills and health literacy must be given to resettlement and integration practices and policies to improve refugee health and wellbeing.

## Study Limitations

This study has many strengths, including being the first collection of literacy and health literacy data at the individual-level for a diverse refugee community that has rarely been studied. One limitation is that English proficiency and health status are self-reported; people may not be fully aware of their language limitations or health issues. Second, the validity and meaning of self-reported health literacy skills may also vary across refugee groups (Kandula et al., 2007). Third, purposive convenience sampling was used in this study, although we attempted to increase generalizability by recruiting at multiple clinics and refugee agencies throughout the community. Finally, our sample size was small (*N* = 136).

## Figures and Tables

**Table 1:** Study Demographics (*N* = 136)^a^

**Variable**	***n* (%)**

Sex	
Male	47 (34.6%)
Female	89 (65.4%)

Married	85 (62.5%)

Country of Origin	
Democratic Republic of Congo	37 (27.2%)
Burma	27 (19.9%)
Afghanistan	10 (7.4%)
India	10 (7.4%)
Iraq	9 (6.6%)
Ethiopia	9 (6.6%)
Tanzania	7 (5.1%)
Syria	6 (4.4%)
Burundi	5 (3.7%)
Central African Republic, Pakistan, Somalia, Trinidad	8 (5.9%)
Bhutan, Zaire, Iran, Liberia, Nepal, Senegal, Sudan, Thailand, Virgin Islands	8 (5.8%)

Employed	56 (41.2%)

Years in United States	
Less than 2.5 years	72 (52.9%)
More than 2.5 years	64 (47.1%)

Health status	
Poor/fair	56 (41.2%)
Good/very good/excellent	77 (56.6%)
Do not know	3 (2.2%)

Has health insurance	40 (29.4%)

Low health literacy	90 (66.2%)

Limited English proficiency	78 (57.4%)

Note.

a Age range: 18–65 years.

**Table 2:** Correlation of Literacy Variables and Health Status

**Variables**	**Health Literacy**	**English Proficiency**	**Length of Time in United States**	**Health Status**
Health literacy	-	0.77^*^	0.34	0.40^*^
English proficiency	0.77^*^	-	0.38^*^	0.37^*^
Length of time in United States	0.34	0.38^*^	-	0.17
Health status	0.40^*^	0.37^*^	0.17	-

Note.

* *p* < .05.

**Table 3:** Results of Logistic Regression Modeling: Health Status as a Function of Literacy Variables, Age, and Gender

**Parameter**	**Odds Ratio**	***p***
Health literacy	2.04	.26
English proficiency	0.99	.98
Health literacy/English proficiency	5.08^*^	.02
Length of time in United States	1.46	.45
Age	0.96^*^	.02
Gender	-	.08

Note. The dependent variable (health status) and health literacy, English proficiency, and length of time in the United States are dichotomized. Age is continuous and female is the referent category for gender.

* *p* < .05.
